# Basal Ganglia Dopamine Availability and Immune Activation Interact and Relate to Anhedonia Severity among Youth with Depression

**DOI:** 10.21203/rs.3.rs-8442008/v1

**Published:** 2026-02-12

**Authors:** Iris Ka-Yi Chat, James W. Murrough, Scott Russo, Finnegan Calabro, Beatriz Luna, Kalyan Tripathy, Tina Gupta, Zachary Brodnick, Megan M. Julien, Neil P. Jones, Neal D. Ryan, Helmet T. Karim, Rebecca B. Price, Jihui Diaz, Erika E. Forbes

**Affiliations:** 1 Department of Psychology, University of California, Los Angeles, Los Angeles, CA, USA; 2 Department of Psychiatry, University of Pittsburgh Medical Center, Pittsburgh, PA, USA; 3 Depression and Anxiety Center for Discovery and Treatment, Department of Psychiatry, Icahn School of Medicine of Mount Sinai, New York, NY, USA; 4 Nash Family Department of Neuroscience, Icahn School of Medicine at Mount Sinai, New York, NY, USA; 5 VISN 2 Mental Illness Research, Education, and Clinical Center (MIRECC), James J. Peters VA Medical Center, Bronx NY, USA; 6 Brain-Body Research Insititute, Icahn School of Medicine at Mount Sinai, New York, NY, USA; 7 Department of Psychology, University of Oregon, Eugene, OR, USA; 8 Department of Neuroscience, The Ohio State University, Columbus, OH, USA; 9 School of Nursing, Duke University, Durham, NC, USA

**Keywords:** inflammation, immune, dopamine, reward, motivation, anhedonia

## Abstract

Anhedonia emerges in adolescence, has putative substrates in neural reward and dopamine systems, and is a hallmark of poor course in depression. Psychoneuroimmunology models suggest altered immune and reward systems may jointly underlie its development. When dopamine availability (rather than function) is considered, immune activation may be understood as a moderator amplifying how lower dopamine levels translate into motivational deficits. This cross-sectional study analyzed data from 55 youth with current depression (M_age_=21.4 years; Female=75%). Plasma was assayed for relative quantification of 92 immune proteins, followed by dimensionality reduction via principal component (PC) analysis, forming five PC scores. Dopamine availability was indexed by basal ganglia tissue iron, quantified by MRI R2’, a measure detectable in youth. Anhedonia was assessed using the Snaith–Hamilton Pleasure Scale (SHAPS), thought to capture overall anhedonia, and Positive Valence Systems Scale (PVSS), which provides overall, anticipatory, and consummatory anhedonia scores. PC1 scores—whose loadings indicated broadly distributed immune protein elevations consistent with general immune activation—moderated the relationship between basal ganglia dopamine availability and anhedonia: At higher PC1 levels, lower dopamine availability was linked to greater overall anhedonia (as measured by SHAPS) and anticipatory anhedonia, but not to consummatory anhedonia. Lower PC2 scores were associated with greater PVSS overall and consummatory anhedonia. Analyses examining individual basal ganglia regions suggest potential differences in dopaminergic-immune interactions across these regions, with effects most consistently observed in the putamen. Findings highlight nuanced neuroimmune pathways underlying anhedonia components in youth and support inflammation-based models of depression emphasizing dopaminergic-immune interplay.

## Introduction

Depression is a major public health concern given its high prevalence, recurrence, and status as a leading cause of disability worldwide (1,2). Despite this burden, approximately 30–50% of individuals respond poorly to available treatments, including antidepressant medications (3,4). Youth, in particular, face an increased risk for first-onset depression and are in a vulnerable period of neural and immune system development (5,6). Depression beginning in youth often persists into adulthood, conferring greater risk for recurrence and severe functional impairment (7,8). A symptom that often first emerges during this period—and that predicts a worse clinical course, suicidality, treatment resistance, and greater severity of depression—is anhedonia (9,10), defined as a lack of pleasure or reactivity to pleasurable stimuli (11). As a key factor in the nature and course of depression and related psychopathology, anhedonia has remained a substantial clinical challenge despite growing attention and research effort over the last five decades (12). As such, it is essential to deepen our understanding of the underlying mechanisms that remain inadequately considered in youth, a vulnerable population.

One valuable approach toward understanding the development and mechanisms of anhedonia is to adopt a more granular conceptualization that distinguishes between components potentially driven by distinct mechanisms. Notably, anticipatory and consummatory anhedonia represent different temporal phases of reactivity to pleasurable stimuli: the motivational drive or ‘wanting’ of rewards, and the experience of pleasure or ‘liking’ during reward receipt (13,14). These components involve dissociable neural pathways. Dopaminergic signaling in mesolimbic pathways is more closely linked to anticipatory as opposed to consummatory pleasure (15,16), whereas opioidergic activity has been more strongly associated with consummatory pleasure, with evidence showing that pleasurable experiences are more sensitive to mu-opioid receptor expression and function than to the dopaminergic pathway (17–19). The Research Domain Criteria (RDoC) framework situates anticipatory and consummatory components of anhedonia within the Positive Valence Systems (PVS) domain. PVS processes encompass approach motivation and responsiveness to reward cues and to reward outcomes, and anticipatory and consummatory anhedonia largely map onto these cue- and outcome-related processes. In addition, the PVS domain also includes broader reward-related constructs such as reward valuation and reward learning (11). When conceptualizing overall anhedonia within the PVS framework, it can be understood as reflecting general functioning across the system as a whole.

Beyond parsing anhedonia into components, psychoneuroimmunology models further highlight the importance of immune signaling as a biological context influencing dopamine-mediated reward processes (20,21). These models generally propose mediational relationships, in which immune activation alters dopaminergic function—such as synthesis, receptor binding, or postsynaptic signaling—which in turn leads to anticipatory anhedonia (21–24). However, when dopamine availability rather than dopamine function is considered, immune activation may be better understood as a moderator that amplifies how lower dopamine levels translate into motivational deficits. Mechanistically, lower dopamine availability in dopaminergic receptor–rich regions such as the basal ganglia reduces phasic dopaminergic bursts necessary for reinforcing motivated behavior and signaling the salience of potential rewards. Inflammation, particularly in the periphery, can further impair various aspects of dopaminergic transmission—dampening postsynaptic sensitivity, reducing receptor availability, disrupting receptor binding, or damaging dopamine-producing neurons (see Treadway et al. (24) for a comprehensive review). These changes could render dopamine functionally less efficient in mediating reward motivation, thereby eliciting even greater anticipatory anhedonia. The signaling between the two systems is a two-way street, as reward circuits can also modulate inflammation in the periphery (23,25,26). Over time, this bidirectional connection can form feedback loops, setting the stage for sustained compromised dopaminergic function and low-grade inflammation. Thus, when lower dopamine availability and the presenece of immune activation are jointly considered, greater anticipatory anhedonia would be expected.

In addition to the biological plausibility, the proposed effects are consistent with several functional magnetic resonance imaging (fMRI) studies examining the links between the interaction of inflammation and dopamine-mediated reward-related brain activity and anhedonia. An observational study found that higher inflammatory composite scores amplified the association between corticostriatal functional connectivity and anhedonia severity (27). Several double-blind, randomized, clinical trials on anhedonia (28,28,29) considered both patients’ levels of inflammation and reward-related neural function. Together, these studies demonstrated improvement in motivation-related and anhedonia symptoms in patients with depression and inflammation following interventions targeting dopaminergic function or inflammation (i.e., levodopa, infliximab; (28,28,29)). Although these intervention studies did not measure dopamine availability, the relevant mechanism of change requires an increase in central dopamine availability, thereby supporting the proposed interactive role of dopamine availability and inflammation.

Collectively, the mechanistic rationale and empirical research converge to support immune-dopamine interactions in anhedonia. However, most existing research on these neuroimmune processes have been conducted in adult samples. This leaves our understanding unclear on whether the interaction effects would similarly be observed in youth, an under-examined population yet characterized by high risk for anhedonia and developing reward-related brain and immune systems that are susceptible to changes in neuroimmune processes. A methodological challenge to execute this research is that, historically, positron emission tomography (PET) was the primary method for assessing dopamine availability. The high cost and health risks (due to radiation exposure) of PET constrain its use in youth populations. More recently, MRI-based measures of tissue iron concentration have emerged as validated, non-invasive proxies for tracking dopamine levels during development, based on the rationale that iron supports dopamine metabolism (30). Tissue iron serves as a proxy for regional differences in dopamine synthesis capacity, and not for other aspects of dopaminergic function including phasic dopamine release at the synapse and receptor binding potential. Leveraging this non-invasive measure can help determine whether the putative reward–related neuroimmune mechanisms apply to youth.

This study examined the moderating role of peripheral immune function in the link between dopamine availability within the basal ganglia and components of anhedonia among youth with current depression. Drawing on existing conceptual and empirical research, both immune and dopaminergic processes are putatively relevant to anticipatory anhedonia. Furthermore, because immune activation can impair dopaminergic efficiency, we expected that greater levels of immune activation would strengthen the association between lower dopamine availability and greater anticipatory, but not consummatory, anhedonia. Effects on overall anhedonia were additionally examined using two measures, one reflecting the classical conceptualization of anhedonia as diminished hedonic capacity (Snaith–Hamilton Pleasure Scale; (31)), and the other operationalizing anhedonia in terms of functioning within the Positive Valence Systems domain—specifically responsiveness and valuation of rewards (Positive Valence Systems Scale (11,32–34)). Overall anhedonia was indexed using total scores from both measures, whereas anticipatory and consummatory anhedonia were assessed specifically using the two PVSS subscales, which corresponds to anticipation of potential rewards and responsiveness to reward receipt, respectively (34). Findings from this study can advance the understanding of the interaction effects of immune and reward-related neural function, informing shared and distinct pathways underlying anhedonia component, and ultimately novel, integrative, and precise intervention targets among youth.

## Methods

### Participants

Fifty-five youth with depression (15–25 years; *M*_*age*_=21.3 years, *SD*=2.18) from the Pittsburgh area completed the current study as part of the larger project, Anhedonia, Development, and Emotions: Phenotyping and Therapeutics (ADEPT), which investigated phenotyping and treatment of anhedonic depression in youth.

All participants had current depressive disorder (i.e., meeting diagnostic DSM-5 criteria through the Structured Clinical Interview for DSM-5 (SCID-5 (35)) and scored 12 or above on the Montgomery-Åsberg Depression Rating Scale (MADRS). Severity of anhedonia (measured by Snaith–Hamilton Pleasure Scale) varied, with moderate-severe anhedonia oversampled (*M*=35.11, *SD*=6.19, *Range*=25–50). Participants were free of lifetime psychosis, bipolar disorder, moderate to severe substance use disorder over the past six months; serious, unstable cardiovascular or neurological disorders; inflammatory disorders; history of brain injury with loss of consciousness; current acute infection; past-month illicit stimulant use; pregnancy; and MRI contraindications (e.g., metal implants). Body mass index was not an exclusionary criterion; however, physical limitations of the MRI scanner used in the larger study imposed practical constraints on body size/shape. Pharmacotherapy and psychotherapy were allowed.

All procedures were approved a priori by the University of Pittsburgh Pitt Protocol Review Online (PittPRO), and written informed consent was obtained from all participants (with assent from those under 18 years old and consent from a parent or guardian).

### Measures

#### Snaith-Hamilton Pleasure Scale (SHAPS)

The SHAPS is a self-report measure that assesses the severity of anhedonia (as defined by hedonic capacity (31)). This measure follows a 4-point Likert scale, with higher score reflecting more severe overall anhedonia.

#### Positive Valence System Scale

The Positive Valence System Scale (PVSS) is a self-report measure designed to assess functioning of the RDoC Positive Valence System (PVS) constructs, that is, Reward Responsiveness and Reward Valuation (34), contrasting with the SHAPS measure, which primarily is intended to capture the consummatory aspect of Reward Responsiveness (31). Anticipatory and consummatory anhedonia were measured using the Reward Anticipation and Initial Responsiveness subscales, respectively. The PVSS scales use a 9-point Likert scale, with lower scores reflecting higher severity of anhedonia. In this sample, the PVSS total, anticipatory anhedonia, and consummatory anhedonia scores were moderately associated with the SHAPS score (i.e., *r*_*total*_=−.63, *r*_*anticipatory*_=−.62, and *r*_*consummatory*_=−.65, respectively; Table 1).

#### The Structured Clinical Interview for DSM-5 (SCID-5)

The SCID-5 (35) is a semi-structured clinical interview designed to assess current and past diagnostic history of psychopathology. This interview was conducted by trained ADEPT research assistants and postdoctoral fellows.

#### Montgomery-Åsberg Depression Rating Scale (MADRS)

The Montgomery-Åsberg Depression Rating Scale (MADRS (36)) is a 10-item interviewer-rated questionnaire assessing several depressive symptoms. This measure used a 7-point Likert scale, with higher scores indicating more severe depression.

#### Dopamine Availability in the Basal Ganglia

R2’, a quantitative MR-based measure (37), was used to assess basal ganglia tissue iron concentration as a proxy of presynaptic dopamine function (30). R2’ reflects the reversible transverse relaxation rate (1/T2’), calculated across the basal ganglia and within each of the subregions (i.e., caudate, putamen, pallidum, and nucleus accumbens) as the difference between the effective R2* (1/T2*) and irreversible R2 (1/T2) relaxation rates (37).

Following previous work by our group (30), R2* data were acquired using a multi-echo gradient echo (mGRE) sequence (TE=3.8, 8, 18, 23 ms; TR=1110 ms, flip angle=25°, FoV=240×240 mm, 40 slices with 0.938×0.938 mm in place resolution and 3 mm spacing). R2 data were acquired using a multi-echo turbo spin echo (mTSE) sequence (TE=12, 99, 186 ms, TR=10800 ms, FoV=240×240 mm, 40 slices with 0.938×0.938 mm in place resolution and 3 mm spacing). R2 and R2* were estimated using a quadratic penalized least squares (QPLS) method (following Funai et al., IEEE Trans. Med. Imaging, 2008) to fit multi-echo data to a mono-exponential decay model (see Larsen et al. (30) for full description). R2* and R2 images were registered to MNI space using AFNI (Cox, 1996), using an affine registration to the anatomical, and non-linear warp to MNI space based on the registration of the anatomical to the MNI template. Estimates of R2’ were derived by subtracting R2 from R2* estimates. All R2’ images were visually assessed by trained research personnel for motion, shimming, susceptibility, or registration artifacts. Scans with visually identifiable artifacts were excluded from all iron analyses (*n*=15).

Given the putative functional distinctions among basal ganglia subregions (i.e., caudate, putamen, pallidum, and nucleus accumbens), secondary analyses were conducted in which each subregion replaced the combined basal ganglia signal for any effects observed at the whole–basal ganglia level.

#### Assay for Immune-Related Proteins

Blood was collected into a BD vacutainer tube via venipuncture. To avoid confounding effects, participants were instructed to avoid alcoholic beverage overnight prior to the blood draw. Thirty minutes later, the collected samples were centrifuged (15 minutes at 3000 RPM) to separate plasma, which were aliquoted and then stored in a −80°C freezer until the time of analysis. Plasma samples were assayed and analyzed for levels of immune-related proteins, using Olink Target 96 Inflammation panel (Olink Bioscience, Uppsala, Sweden), according to the manufacturer’s instructions, at the Human Immune Monitoring Center of the Icahn School of Medicine at Mount Sinai. This panel measures 92 target proteins (principally chemokines, interleukins, growth factors, TNF-related molecules, other inflammation-related proteins). The assay provides relative quantification of these targets, expressed as normalized protein eXpression (NPX) unit on a log2 scale. Higher NPX value indicates higher protein concentration. Following established procedures, if a specific target in the panel yielded values for which ≥ 25% of the samples were lower than the limit of detection (LOD) or did not pass quality control, the target overall was excluded from the analysis. This results in 70 immune proteins included for further analyses (see Supplementary Table 1 for descriptive statistics of included proteins).

### Principal Component Analysis for Immune Proteins

To reduce the dimensionality of the immune protein data, a principal component analysis was conducted following prior work (38). Individual outliers (i.e., standard deviations > 3 from the group mean) were substituted by multiple imputations. The optimal number of principal components to retain was determined using a combination of criteria, including inspection of the scree plot and results from Horn’s parallel analysis (39), where components with eigenvalues greater than those derived from randomly simulated data are considered optimal for retention. Both approaches indicate inflection after the fifth component (see Results for details). Accordingly, the first five principal components were retained, collectively explaining approximately 55% of the total variance. We were interested in the dimension reflecting general immune activation, characterized by positive loadings across numerous immune proteins, and a principal component score for each participant was extracted and used for the primary analyses. Exploratory analyses were completed using the scores of the remaining principal components retained.

### Statistical Analyses

Multiple linear regressions were conducted to examine the interaction effects of dopamine availability in the basal ganglia (as the independent variable) and immune PC scores (as the moderators) on the severity of overall, anticipatory, and consummatory anhedonia (as the dependent variables). If the interaction effects were significant, the Johnson-Neyman procedure was used to probe the range of immune PC scores at which dopamine availability was significantly associated with anhedonia. In addition, a secondary analysis was conducted to probe effects within subregions of basal ganglia (i.e., caudate, nucleus accumbens, pallidum, putamen). Exploratory analyses repeated the above models using the remaining immune PC scores retained. Covariates were sex assigned at birth, body mass index, age, and current psychotropic medication use (yes vs. no; e.g., antidepressants, mood stabilizers, antipsychotics, sedative hypnotics, and benzodiazepines). These covariates were included given their known associations with immune and/or brain functions (40–42). Models also adjusted for the severity of depressive symptoms (excluding anhedonia) to help isolate effects specific to anhedonia. All analyses were conducted in R software. Sample characteristics and correlations among study variables are displayed in Table 1.

## Results

### Preliminary Analyses

#### PCA Results: Immune Activation

**Fig. 1:**
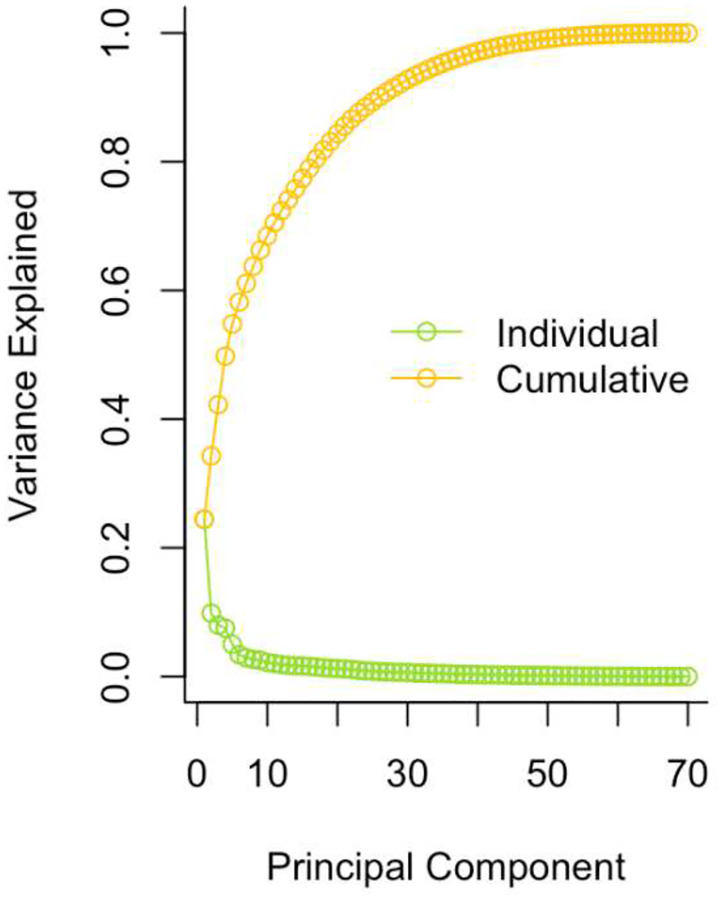
Principal Component Analysis Results: Immune Activation. Scree plot illustrating the principal component results based on the 70 immune-related proteins. The first five principal components made up 55% of total variance, accounting for 24%, 10%, 8%, 8% and 5% of variance, respectively.

The scree plot in Figure 1 shows the PCA results based on the 70 immune-related proteins, indicating that the first five principal components made up 55% of total variance, accounting for 24%, 10%, 8%, 8%, and 5% of variance, respectively. Loadings for the 70 immune proteins in the five principal component scores are listed in the supplementary material (Supplementary Table 2), sorted by absolute value from highest to lowest. Because nearly all immune proteins (68 of 70) had positive loadings on the first principal component (PC1) and the loadings were generally modest in magnitude (largest=.193), this component was interpreted as reflecting broadly distributed, low-to-moderate positive contributions across markers, consistent with *general immune activation*—the construct of primary interest in this study. Thus, the primary analyses were conducted using the PC1 score as provided below, whereas exploratory analyses were conducted for PC2, PC3, PC4, and PC5 scores.

### Primary Analyses

#### General Immune Activation (PC1) and Anhedonia

Main effects were found for higher immune activation (PC1 score) on more severe SHAPS overall anhedonia (*B*=3.483, *SE*=1.310, *p*=.011, *ΔR*^*2*^=.105) and PVSS anticipatory anhedonia (*B*=−2.859, *SE*=1.104, *p*=.013, *ΔR*^*2*^=.085). Immune activation was unrelated to PVSS overall anhedonia (*B*=−.004, *SE*=.047, *p*=.931, *ΔR*^*2*^<.001) and PVSS consummatory anhedonia (*B*=.067, *SE*=.209, *p*=.751, *ΔR*^*2*^=.002).

#### Dopamine Availability in Basal Ganglia and Anhedonia

We did not detect main effects for dopamine availability within the basal ganglia on SHAPS overall anhedonia (*B*=−.387, SE=.341, *p*=.262, *ΔR*^*2*^=.019), PVSS overall anhedonia (*B*=.111, *SE*=.082, *p*=.179, *ΔR*^*2*^=.031) or PVSS consummatory anhedonia (*B*=.378, *SE*=.364, *p*=.305, *ΔR*^*2*^=.020), except for PVSS anticipatory anhedonia (*B*=.623, *SE*=.287, *p*=.035, *ΔR*^*2*^=.060).

#### Interaction of Dopamine Availability in Basal Ganglia and Immune Activation in Relation to Anhedonia

**Fig. 2.**
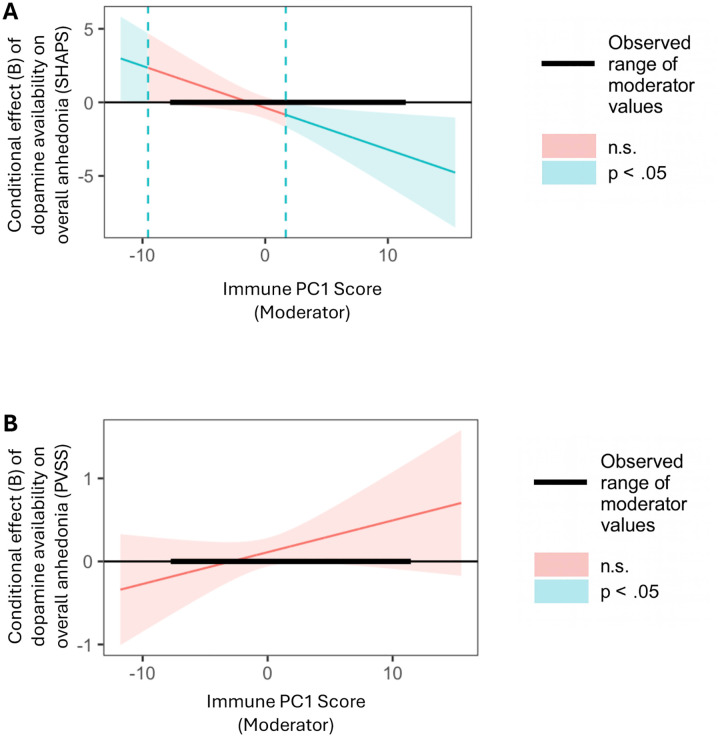
Conditional effects of dopamine availability in the basal ganglia on overall anhedonia by levels of general immune activation. Johnson-Neyman plots showing regions of statistical significance for the moderation effect of general immune activation (Principal Component 1) on the association between dopamine availability in the basal ganglia and overall anhedonia, as measured by the Snaith–Hamilton Pleasure Scale (SHAPS; A) and the Positive Valence Systems Scale (PVSS; B). Blue and red shaded areas denote the levels of general immune activation at which the conditional effect was significant (*p* <.05) versus non-significant (n.s.; *p* ⩾ .05), respectively. The thick black segment indicates the observed range of moderator values (i.e., immune PC1 score) in the sample; effects outside this range represent extrapolations beyond the observed data. **A.** At higher level of general immune activation, lower dopamine availability in the basal ganglia was associated with higher overall anhedonia (as measured by the SHAPS). **B.** The moderation effect was not observed when overall anhedonia was measured by the PVSS.

There was an interaction effect between dopamine availability in the basal ganglia and immune activation on SHAPS overall anhedonia (*B*=−.299, *SE*=.109, *p*=.009, *ΔR*^*2*^=.111), such that for participants in the top 68.97% of levels of immune activation, lower dopamine availability was associated with more severe SHAPS overall anhedonia (Figure 2A). We did not detect an interaction effect for PVSS overall anhedonia (*B*=.042, *SE*=.026, *p*=.113, *ΔR*^*2*^=.042; Figure 2B).

**Fig. 3.**
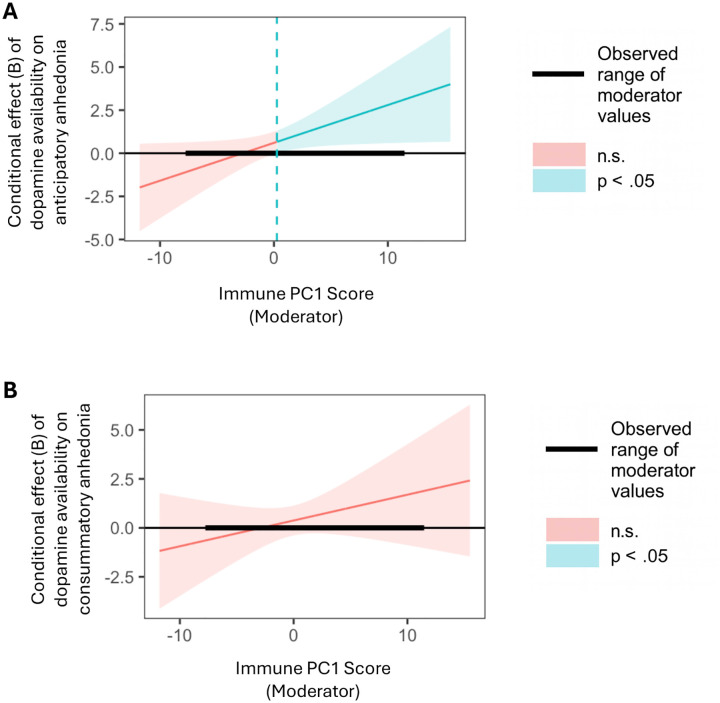
Conditional effects of dopamine availability in the basal ganglia on anhedonia components by levels of general immune activation. Johnson-Neyman plots showing regions of statistical significance for the moderation effect of general immune activation (Principal Component 1) on the association between dopamine availability in the basal ganglia and components of anhedonia (i.e., anticipatory anhedonia [A] and consummatory anhedonia [B]; as measured by the subscales of Positive Valence Systems Scale). Blue and red shaded areas denote the levels of general immune activation at which the conditional effect was significant (*p* <.05) versus non-significant (n.s.; *p* ≥ .05), respectively. The thick black segment indicates the observed range of moderator values (i.e., immune PC1 score) in the sample; effects outside this range represent extrapolations beyond the observed data. **A.** At higher level of general immune activation, lower dopamine availability in the basal ganglia was associated with higher anticipatory anhedonia. **B.** The moderation effect was not observed for consummatory anhedonia.

There was also an interaction effect for PVSS anticipatory anhedonia (B=.235, *SE*=.092, *p*=.014, *ΔR*^*2*^=.084), such that for participants in the top 58.62% of levels of immune activation, lower dopamine availability was significantly associated with more severe PVSS anticipatory anhedonia (Figure 3A). We did not detect an interaction effect for PVSS consummatory anhedonia (*B*=.144, *SE*=.116, *p*=.220, *ΔR*^*2*^=.028; Figure 3B).

### Secondary and Exploratory Analyses

Secondary analyses were conducted to probe effects within subregions of basal ganglia for any significant findings identified in the primary analyses, and exploratory analyses examined associations with the four other immune principal component scores. Full details of results are reported in the Supplementary Material.

Dopamine Availability X Immune PC1 interaction effects were present for several basal ganglia subregions for SHAPS overall anhedonia (caudate, pallidum, putamen) and anticipatory anhedonia (pallidum, putamen).

Lower PC2 scores were associated with overall PVSS anhedonia and consummatory anhedonia, and not anticipatory anhedonia or SHAPS overall anhedonia. No interaction effect for PC2 was detected.

There was a Dopamine Availability X Immune PC5 interaction effect on SHAPS overall anhedonia. For participants in the bottom 32.76% of PC5 scores, lower dopamine availability in the basal ganglia was associated with greater SHAPS overall anhedonia. Follow-up analyses indicated that the effect was observed in putamen. There were no effects with the remaining PC scores.

## Discussion

This study examined whether peripheral immune processes and naturally occurring dopamine availability in the basal ganglia, alone and in interaction, were associated with anhedonia among youth with current depression. As hypothesized, we found that higher self-reported anhedonia was associated with higher scores on immune PC1, which reflected broadly distributed, low-to-moderate positive loadings across immune proteins consistent with a global activation pattern. Likewise, dopamine availability was associated with anticipatory anhedonia (14). Aligned with the conceptual and empirical literature of immune pathways in anhedonia (20,43), we also found that general immune activation amplified the effect of lower dopamine availability in the basal ganglia on greater anhedonia severity. Moreover, consistent with prior research suggesting that mechanisms of anhedonia vary by its components (14,44), the associations of immune processes and dopamine availability with anhedonia differ based on anhedonia components. In addition, the exploratory analyses preliminarily point to possible differences among subregions in how dopamine availability interacts with immune processes, with effects most consistently observed in putamen, although replication in larger samples will be necessary to confirm these patterns.

The robust amplification effects between low dopamine availiablity and general immune activation detected for greater anticipatory anhedonia as compared to consummatory anhedonia aligns with the theoretical rationale and neurobiological research suggesting that immune activation is relevant to shifting the motivational state and the function in dopaminergic pathways subserving approach motivation (21,24,45). By showing that distinct neural and immune pathways, separately and interactively, may characterize different components of anhedonia, these findings underscore the risk of aggregating anhedonia into a unitary construct without examining or conflating its components (13,14,44). Such lumping can, from an analytical standpoint, dilute true effects and obscure pathway-specific associations. From a precision medicine perspective, failing to disaggregate separate components of anhedonia when examining pathophysiology can increase the risk of mismatching mechanisms with symptom presentations and thus generating imprecise treatment targets.

Notably, interaction effects were detected for overall anhedonia when measured by the SHAPS, but not by total score on the PVSS, although both are expected to capture the same construct. The association between immune activation and SHAPS scores aligns with prior findings, both cross-sectionally in adolescent samples with diverse psychiatric conditions (46) and longitudinally in adolescents with depressive disorders (47). Neither study included the PVSS, however, and therefore could not determine whether the absence of effect was specific to PVSS total or reflected a broader measurement-related discrepancy. One possible explanation for this discrepancy is that the SHAPS, widely considered a gold-standard anhedonia measure, was developed based on the classical definition of anhedonia as a lack of pleasure (31) and, as a result, is often interpreted as indexing consummatory anhedonia. Yet this interpretation appears unlikely, as significant effects were observed for the SHAPS but not the PVSS consummatory subscale, which was designed to capture variability in hedonic response to recipt of reward. Moreover, SHAPS scores were moderately correlated with PVSS consummatory anhedonia (*r*=−.65) and in similar magnitude with PVSS total (*r*=−.63), suggesting that the SHAPS captures anhedonia more broadly than just consummatory anhedonia, while remaining partially dissociable from PVSS total.

A more plausible explanation for this discrepancy lies in linguistic differences between the measures. Although SHAPS items were framed as assessing hedonic capacity over the past few days, they are phrased hypothetically (i.e., “*I would* …”), which can introduce future-oriented and conditional framing and bias responses towards anticipatory pleasure or motivational appraisal, blurring anticipatory versus consummatory anhedonia assessment. In contrast, the PVSS anchors responses in recent actual experience, allowing its consummatory subscale to more precisely capture hedonic capacity (34). Thus, the SHAPS effect may reflect unintended inclusion of anticipatory components. Because PVSS total represents the broad construct spanning general PVS deficits beyond anticipatory and consummatory dimensions, the null result further suggests that collapsing across components may attenuate associations if some PVS components are less relevant to the processes. Such aggregation may obscure component-specific effects, underscoring the value of targeted measures that capture distinct anhedonia components in future research (11,14,48).

Our exploratory analyses additionally found effects using other immune components. Specifically, lower immune PC2 scores—characterized by higher positive loadings on 4E-BP1, AXIN1, SIRT2, STAMBP, and CASP-8, and higher negative loadings on TNF-β and CX3CL1—was associated with more PVSS overall anhedonia and consummatory anhedonia, and not SHAPS overall anhedonia or anticipatory anhedonia. In addition, an interaction effect indicated that, at lower immune PC5 scores (characterized by higher positive loadings on DNER, CX3CL1, TNF-β, and ADA, and higher negative loadings on CD8A, IL-6, OSM, and CCL23), lower basal ganglia dopamine availability was associated with greater SHAPS overall anhedonia. No effects were observed for immune PC3 (higher positive loadings on TWEAK, and EN-RAGE, and negative loadings on ST1A1, SIRT2, AXIN1) or PC4 scores (higher positive loadings on TRAIL, uPA, CD8A, and TNF, and negative loadings on ST1A1). These findings collectively underscore that immune processes are not monolithic. Rather, distinct constellations and contrasts among immune processes (e.g., adaptive or regulatory pathways vs. proinflammatory activity) may yield diffrent clinical implications across specific symptom dimensions. However, most prior research on immune function in relation to psychopathology has focused on elevated levels of individual markers or small cyokine panels—an approach that may overlook broader patterns of immune activity relevant to symptom heterogeneity. In contrast, our analyses leveraged a large-scale multiplex assay—an innovative approach that enabled comprehensive immune profiling across numerous markers. This approach provided substantially greater granularity than traditional single-marker methods and allowed immune pathways (e.g., inflammatory and antiviral pathways) to be examined both independently and jointly, offering a more nuanced understanding of how immune function relates to psychopathology. Given the exploratory nature of these analyses, future research deriving factors from large sets of immune markers should replicate this effort using a confirmatory analytical approach.

Given that most studies on brain-immune pathways and related interventions have been conducted in adults, a critical question has been whether such findings extend to other developmental stages, as developmental context may shape how immune, dopaminergic, and affective systems interact, and, in turn, how these interactions confer risk for anhedonia (6). For youth, this context is characterized by ongoing maturation of mesocorticolimbic dopaminergic pathways and immune signaling processes. Because these systems are highly plastic and mutually influential during this period (6), disruptions in one are likely to affect the other, thereby heightening risk for anhedonia. However, whether empirical research supports this theoretical notion remains unclear, as this area of research is generally underdeveloped in youth. The current study begins to bridge this gap, providing preliminary support for the potential generalizability of prior mechanistic and interventional findings to a younger developmental stage. Further research investigating brain-immune mechanisms underlying anhedonia within developmental contexts will be valuable for informing early, targeted, and developmentally sensitive interventions for this vulnerable age group.

This study integrated multiple levels of analysis from self-report measures to biological and neural indices, providing a comprehensive understanding of pathways underlying anhedonia. It also addressed a gap in the literature by examining interactions between two systems typically studied independently: reward-related (dopaminergic) neural function and immune activation. Most prior work has hypothesized *mediational* pathways in which immune activation impairs dopaminergic function to induce anhedonia, a question best tested in longitudinal designs, especially in youth undergoing rapid maturation. The current findings instead highlight the complementary value of an moderation model, wherein immune activation may create a biological context that renders available dopamine less effective in supporting reward-related function. Notably, this is among the first studies to apply the tissue iron technique, an advance that allows for greater insight into neurochemical mechanisms of psychopathology in youth, which is typically under-examined due to the limitations of PET and other molecular imaging approaches for younger populations. Consistent with expectations, the dopamine variable was detectable in the sample even though tissue iron is deposited during development. The cross-system focus, alongside consideration of methodological issues (e.g., lumping symptoms) may help explain inconsistencies across prior studies (49,50).

Nevertheless, several limitations should be considered when interpreting the findings. First, given the cross-sectional and observational design, the findings are correlational and do not establish causality. Although direct manipulation of dopaminergic function is not feasible, experimental inflammation paradigms (e.g., immune challenge) and pharmacological tools such as dopaminergic agents may provide complementary approaches to strengthen causal inference. Second, while this study’s decision to limit recruitment to youth is a strength in terms of understanding development and early course of anhedonia, it also restricts the ability to draw conclusions about the specificity and generalizability of the findings across the lifespan. Third, although tissue iron provides indirect measure of dopamine availability, other aspects of dopamine function (e.g., synaptic release, receptor availability) cannot be concluded. Such processes would require PET, whose elective use for research purposes would not be ethically feasible in younger participants. Fourth, although prior research has documented sex-related variations in the current constructs of interest (51–53), we could not adequately examine potential sex differences in the observed effects due to unequal sex distribution and limited sample size. Future work with larger and more balanced samples is needed to test these differences and determine whether they also apply to youth, whose neural, immune, and hormonal systems are undergoing rapid and dynamic maturation (6). Finally, other key reward processes relevant to anhedonia, particularly reward learning, were not assessed, which limited possible conclusions about anhedonia and reward processing alterations.

Taken together, our findings suggest that different neural and immune pathways may be uniquely associated with different components of anhedonia in youth and underscore the importance of jointly assessing reward and immune processes when studying anhedonia. This integrated approach holds promise for informing more targeted treatments for this debilitating symptom.

## Supplementary Material

Supplementary Files

This is a list of supplementary files associated with this preprint. Click to download.

- ADEPTsupp2025.12.23.docx

Supplementary information is available at Molecular Psychiatry’s website.

## Figures and Tables

**Table 1.** Sample characteristics and bivariate correlations of study variables (N = 55).

Variable	*M / n*	*SD / %*	Range	1	2	3	4	5	6	7	8	9	10	11	12
Sex															
Female	41	74.5%													
Male	14	25.5%													
Race															
Asian	7	12.7%													
Black / African American	5	9.1%													
Multiple Races	1	1.8%													
White	42	76.4%													
Psych Meds															
Yes	41	74.5%													
No	14	25.5%													
1. Age (Years)	21.35	2.18	15–25												
2. BMI	26.14	7.11	17.2–43.2	.20											
3. DA	11.26	2.35	6.28–15.9	.24	.23										
4. PC1	−0.05	4.25	−7.54–11.2	.11	.20	.05									
5. PC2	−0.18	2.65	−6.04–4.81	−.16	−.07	−.19	−.02								
6. PC3	0.26	2.44	−4.14–7.74	.06	−.23	.02	.14	−.13							
7. PC4	−0.06	2.22	−4.72–5.77	.16	−.33*	−.04	.03	.02	−.03						
8. PC5	0.11	1.84	−4.05, 4.21	.04	−.38**	−.13	−.10	−.15	.01	.14					
9. SHAPS	35.11	6.19	25.0–50.0	.26	.13	−.05	.11	−.25	−.09	−.03	.18				
10. PVSS Total	5.08	1.40	2.33–7.95	−.18	−.07	.13	−.17	.36**	−.12	−.08	.02	−.63**			
11. Anticipatory Anhedonia	14.80	5.62	3–26	−.21	−.02	.17	−.20	.25	−.02	−.03	.00	−.62**	.80**		
12. Consummatory Anhedonia	21.45	5.92	9–35	−.11	−.05	.09	−.06	.34*	−.09	−.02	.01	−.65**	.89**	.67**	
13. MADRS Total	29.04	7.81	14–46	.16	.05	.09	.27*	−.18	.12	.02	.08	.36**	−.36**	−.46**	−.32*

*Note*.

* *p*<.05,

** *p*<.01,

*** *p*<.001.

Abbreviations: Psych Meds, Psychotropic medication use; BMI, Body mass index; DA, Dopamine availability in the basal ganglia; PC, principal component; SHAPS, Snaith–Hamilton Pleasure Scale; PVSS, Positive Valence Systems Scale; MADRS, Montgomery–Åsberg Depression Rating Scale.
